# Effects of Prey Distribution and Heterospecific Interactions on the Functional Response of *Harmonia axyridis* and *Aphidius gifuensis* to *Myzus persicae*

**DOI:** 10.3390/insects11060325

**Published:** 2020-05-26

**Authors:** Xing-Lin Yu, Rui Tang, Peng-Liang Xia, Bo Wang, Yi Feng, Tong-Xian Liu

**Affiliations:** 1 State Key Laboratory of Crop Stress Biology for Arid Areas, and Key Laboratory of Integrated Pest Management on Crops in Northwestern Loess Plateau of Ministry of Agriculture, College of Plant Protection, Northwest A&F University, Yangling 712100, China; yuxinglin92@nwafu.edu.cn (X.-L.Y.); 2017060087@nwafu.edu.cn (R.T.); wangbo.1010@nwafu.edu.cn (B.W.); 2 Hubei Tobacco Company Enshi State Co., Ltd., Enshi 445000, China; xiangpengliang@163.com

**Keywords:** prey distribution, interspecific interactions, intraguild interactions, biological control

## Abstract

Natural enemy guilds normally forage for prey that is patchily distributed simultaneously. Previous studies have investigated the influence of conspecific interactions and prey distribution on the functional response of natural enemies. However, little is known about how prey distribution and heterospecific interactions between natural enemies could affect their foraging efficiency. We examined the effects of prey distribution (aggregate and uniform) and heterospecific interactions on the functional response of a predator, *Harmonia axyridis* (Pallas) (Coleoptera: Coccinellidae) and a parasitoid, *Aphidius gifuensis* Ashmead (Hymenoptera: Braconidae) to the green peach aphid, *Myzus persicae* (Sulzer) (Hemiptera: Aphididae). Type II functional responses were observed in all experiments. Functional response curves of single *H. axyridis* or *A. gifuensis* were higher in the aggregate treatment than in the uniform treatment when aphid densities were between 40–180 or 70–170, respectively. When comparing between aggregate and uniform treatments with the heterospecific enemy occurrence, no differences were found in the parasitism efficiency of *A. gifuensis*, while *H. axyridis* consumed more aphids in the aggregate treatment than in the uniform treatment when aphid densities were between 50–230. The functional response of individual *H. axyridis* was not affected by *A. gifuensis* under two aphid distributions. However, the functional response of a single *A. gifuensis* and the treatment when *A. gifuensis* concurrently with *H. axyridis* overlapped in uniform treatment of above approximately 150 aphids. Our results indicate that the predation rate of *H. axyridis* was affected by aphid distribution, but was not affected by heterospecific interactions. The parasitism rate of *A. gifuensis* was affected by aphid distribution, and by heterospecific interactions in both the aggregate and uniform treatments. Thus, to optimize the management efficiency of *M. persicae*, the combined use of *H. axyridis* and *A. gifuensis* should be considered when *M. persicae* is nearly uniformly distributed under relatively high density.

## 1. Introduction

Biological control by natural enemies is an environment-friendly and effective approach in regulating pest population, and it has received increasing research interest and has long been applied as part of integrated pest management (IPM) strategies [1,2,3]. In most ecosystems, pest species are often associated with multiple natural enemies, and more and more biological control programs have used more than one species of natural enemies [4,5,6]. However, the effect of multiple enemies in regulating prey populations cannot be predicted simply as an additive outcome from the evaluation of the independent effects of each natural enemy [7,8,9]. When combined, multiple enemies are involved in complex interactions, such as predator interference, cannibalism, parasitoid avoidance behavior, and intraguild interactions [6,10,11,12,13]. In such cases, the heterospecific interactions among the foraging enemies may reduce their per capita search activity and attack efficiency at a given host density. However, few studies included the consequences of these interactions on the control efficiency of natural enemies when sharing the same resources in multiple enemy systems [14,15]. 

Evaluating the functional response, which is the relationship between prey density and the number of prey killed by a natural enemy, is a common method to interpret enemy-pest interactions [16,17,18,19]. The functional response could be used to evaluate population dynamics and the prey suppression ability of an agent [17,20,21], and therefore it may provide insight into the mechanisms of enemy-prey interactions and how natural enemies affect pest populations [22,23]. 

In multiple enemy systems, previous studies on the functional response of single enemy species on prey populations have mostly been conducted under simple experimental arenas [14,15]. However, most prey populations are patchily distributed in the field [24,25,26]. Prey distribution could affect the foraging outcome of a natural enemy through affecting the foraging efficiency of the natural enemy that exhibits random searching pattern, because the natural enemy may waste time in patches where prey resources are scarce [27,28]. In addition, previous studies found that natural enemies were attracted to high prey density patches [29,30], and several enemy species are frequently observed exploiting the same patches of prey simultaneously [31,32]. This means that prey aggregation may increase the multiple enemy competition for food-rich patches, which may result in decreased kill rates [33]. Moreover, the dominant predator species can prevent subordinates from foraging effectively when these enemies share prey that are aggregated in a few sites. Therefore, prey aggregation may lead to a further decline in the per capita kill rate [33]. Previous studies have investigated the influence of conspecific interactions and prey distribution on the functional response of *Anax junius* (Odonata: Aeshnidae), in which the per capita kill rate was reduced at low prey: predator ratios in aggregate prey treatment [33]. However, the effects of prey distribution and heterospecific interactions on the functional response of multiple enemies remain largely unknown.

The green peach aphid, *Myzus persicae* (Sulzer) (Hemiptera: Aphididae), is a serious pest of different crops in a wide range of agroecosystems in China and induces severe damage [34]. Moreover, *M. persicae* has various spatial distributions in the field [35]. *Aphidius gifuensis* Ashmead (Hymenoptera: Braconidae) and *Harmonia axyridis* (Pallas) (Coleoptera: Coccinellidae), which are already present in China and are associated with *M. persicae* [36,37]. *Aphidius gifuensis*, a solitary koinobiont endoparasitoid, is a commonly augmented specialist parasitoid in regulating aphids, including *M. persicae* [37,38]. *Harmonia axyridis* is a generalist predator extensively employed as an effective biological control agent against aphids in various cropping systems [39,40]. Studies have investigated the aphid parasitism/predatory capacity of *A. gifuensis* or *H. axyridis* in simple experimental arenas [41,42,43]. In addition, previous studies have evaluated the functional response of *H. axyridis* under different prey distributions [44], or with con- and heterspecific generalist predators in single plant [45]. However, to our knowledge, although there are observations for *H. axyridis* and *A. gifuensis* to overlap and compete for resources in various habitats where *M. persicae* was a pest [46], no previous studies have evaluated how prey distribution and heterospecific interactions could affect their predatory or parasitism efficiency when sharing the same prey species.

In our study, we first investigated the functional response of single *H. axyridis* or *A. gifuensis* to densities of *M. persicae* under different prey distributions. Then, we assessed the effects of prey distribution and heterospecific interactions on the functional response of *H. axyridis* or *A. gifuensis*. We hypothesized that when *H. axyridis* and *A. gifuensis* co-occurred, parasitism rate of *A. gifuensis* will be reduced in the aggregate treatment compared with uniform treatment. A better understanding of the heterospecific interactions between natural enemies under different prey distributions may provide a framework to understand population dynamics of each natural enemy species and guide strategies to increase the efficacy of combining these two natural agents in biological control programs. 

## 2. Material and Methods

### 2.1. Plants and Insects

Chili pepper plants (*Capsicum annuum* L., var. ‘Shulahuojian F1′, six-week-old and around 12 cm in height) were used for rearing aphids or preparing for the experiments. *Myzus persicae*, *A. gifuensis*, and *H. axyridis* were originally collected from chili pepper and cabbage fields at the Experimental Farm (108°04′18″ E, 34°17′52″ N), Northwest A&F University (Yangling, Shaanxi, China) in July 2014. *Myzus persicae* were maintained on chili pepper plants; *A. gifuensis* were originally obtained from *M. persicae* and cultured for at least 12 generations on *M. persicae* on chili pepper plants. *Harmonia axyridis* males and females were paired in Petri dishes (3 cm in diameter) and fed with *M. persicae* to allow mating and oviposition. Newly hatched *H. axyridis* larvae were reared individually in 3-cm Petri dishes and provided with an excess of *M. persicae* daily until they reached the pupal stage. *Harmonia axyridis* adults that emerged within 24 h were isolated and the naïve 2–3-days old unmated female adults were used in all subsequent experiments. All insect colonies and the experiments were maintained in a controlled insectary at 25 ± 1 °C, 65 ± 5% RH and a 16:8 h (L:D) photoperiod.

### 2.2. Functional Response of Single Predators or Parasitoids

For each predator or parasitoid species, we first measured the predation/parasitism rate of a single adult female consuming or parasitizing *M. persicae* on chili pepper plants. Third instar aphid nymphs were used in all experiments to avoid aphid nymph production by adults. To create third instar aphid nymphs, a pepper leaf disc (3 cm in diameter) was placed on the bottom of a small Petri dish (3 cm in diameter) with 1% agar gel. Twenty *M. persicae* adults were introduced in each Petri dish and removed after 24 h. The newborn aphid nymphs were maintained in the Petri dish for another 24 h. Newly molted second-instar nymphs were transferred to new leaf disks and all younger nymphs and the ecdyses were discarded. Aphid nymphs were kept and were used when they grew to the third instar. 

Four chili pepper plants were arranged randomly to each ventilated cage (30 × 30 × 30 cm). Each experimental cage received 4, 8, 16, 32, 64, 128, or 256 third-instar aphid nymphs. Aphids were introduced at the bottom of the stem of the chili pepper plant and allowed to acclimate for 1 h. For the aggregation treatment, all aphids were randomly allocated on one of these plants. However, for the uniform treatment, aphids were divided evenly among four plants. 

Prior to the experiment, unmated adult female *H. axyridis* were individually transferred from the stock culture into Petri dishes (3 cm in diameter) for 24 h to standardize their hunger level. During this time, a water-saturated cotton ball was placed in each Petri dish to provide moisture. For the parasitoid, *A. gifuensis* mummies were collected from plants with a fine camel hair brush and placed in plastic cylindrical cages (12 cm in height by 7 cm in diameter) with 10% honey solution and inspected at regular intervals. All male and female parasitoids that emerged on the same day were placed in new plastic cylindrical cages for 24 h, and the parasitoids were left undisturbed to ensure female mating. Generally, adult males and females normally mated a few hours after emergence [37]. Mated females were used in the experiments.

Then, *H. axyridis* or *A. gifuensis* were placed individually in the center of each cage. After 24 h, predators or parasitoids were removed from the cage. For the predator treatment, the number of aphids consumed was recorded. For the parasitoid treatment, the number of parasitoid mummies was recorded after 10 days. The experiment was replicated 10 times for each treatment. 

### 2.3. Functional Response of Paired Heterospecific Enemies

Starved *H. axyridis* adults and *A. gifuensis* mated females were prepared using the same procedures as described in the relevant sections, and the aphid density and distribution used were the same as described above. One *H. axyridis* adult and one *A. gifuensis* female were introduced to each cage. After 24 h, predators and parasitoids were removed from the experimental cages and the number of aphids preyed upon by predators was recorded. Moreover, the number of parasitoid mummies was recorded after 10 days. The experiment was replicated 10 times for each treatment.

For both the two experiments above, aphids were not replaced during the experiment. In addition, under two aphid distributions, a control treatment without predators and parasitoids was conducted with five replications for each aphid density (4, 8, 16, 32, 64, 128, or 256) to assess natural mortality rates by counting the dead aphids. 

### 2.4. Data Analysis

All analyses were conducted using the statistical software R [47]. To evaluate the type of functional response that best fitted the data in the different experiments, a model selection and hypothesis testing was used [48]. For model selection, a logistic regression of the number of prey killed was used to identify the type of functional responses fitted with the maximum likelihood (ML) procedure. Significant negative or positive linear coefficients from the regression suggest type II or III responses, respectively [48]. When a significant negative linear coefficient from logistic regression was found, the data were then fitted to a type II functional response curve with ML estimation using the random predator Equation (1) [49], which allows for prey depletion:

*N*_e_ = *N*_0_[1 − exp(*aT*_h_*N*_e_ − *aT*] (1)

where *N*_e_ is the number of prey eaten, *N*_0_ is the initial prey density, *a* is the attack rate, *T*_h_ is the handling time, and *T* is the total experimental duration (24 h). To compare functional response fits between natural enemies, the functional response fits were non-parametrically bootstrapped (n = 2000) to generate 95% confidence intervals (CIs) around functional response curves and the associated parameters. Equation (1) was then fitted to the bootstrapped dataset with initial parameter values that were estimated from the original ML estimates. The overlap between confidence intervals indicates that the functional responses and/or the corresponding parameters were not significantly different. Analysis of the observed functional responses modeling was carried out with the ‘frair’ package [50].

Data from trials of single *H. axyridis*, single *A. gifuensis*, and individual *H. axyridis* or *A. gifuensis* in heterospecific combination were analyzed with a generalized linear mixed model (GLMM) (glmer function in the lme4 package) with a binomial distribution. The dependent variables were the number of aphids killed, and the explanatory variables were aphid density and their distributions. Natural enemies tested in each replicate was treated as an observation-level random effect.

## 3. Results

In control treatments, aphid survival in both types of distribution treatment exceeded 98.5% in ventilated cages, and thus aphid’s natural mortality did not attribute to background mortality.

### 3.1. Functional Response of Single Predators or Parasitoids

Significant negative linear terms were detected from logistic regressions for both treatments. This indicated a type II functional response for single *H. axyridis* or *A. gifuensis* (Table 1). The attack rates and handling times of the functional response models were all significant (Table 1).

**Figure 1:**
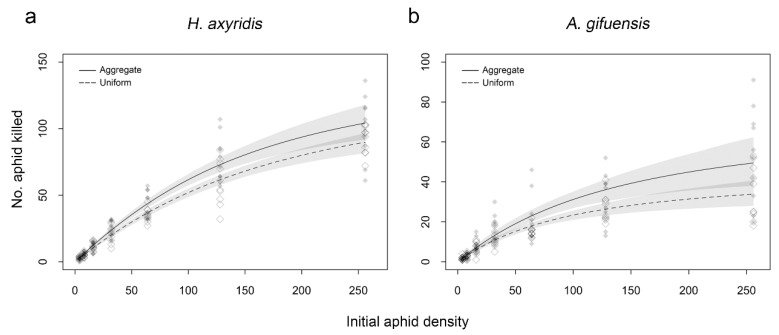
Functional response of single *Harmonia axyridis* (**a**) or *Aphidius gifuensis* (**b**) to densities of *Myzus persicae* under aggregate and uniform treatments. Dashed lines differ in style represent functional response curve with different aphid distribution treatments, while shaded areas are bootstrapped 95% confidence intervals (n = 2000 bootstraps each).

For single *H. axyridis* treatment, we found that aphid density, aphid distribution, and the interaction between aphid density and aphid distribution had a significant effect on aphid consumption by *H. axyridis* adults (Table 2). Functional response curves overlapped at aphid densities below 40 and above 180 between aggregate and uniform treatment (Figure 1a). For single *A. gifuensis* treatment, the aphid density, aphid distribution, and the interaction between aphid density and aphid distribution significantly affected the number of aphids parasitized by female *A. gifuensis* (Table 2). Functional responses of aggregate and uniform treatments overlapped at aphid densities below 70 and above 170 (Figure 1b).

### 3.2. Functional Response of Paired Heterospecific Enemies

Both *H. axyridis* and *A. gifuensis* exhibited type II functional responses when heterospecific enemy species were present (Table 1). The attack rates and handling times of the functional response models were all significant (Table 1).

**Figure 2:**
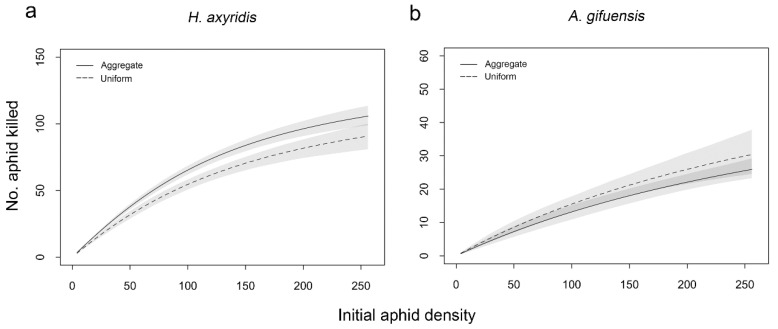
Functional response of individual *Harmonia axyridis* (**a**) or *Aphidius gifuensis* (**b**) to densities of *Myzus persicae* by the presence of heterospecific enemy under aggregate and uniform treatments. Shaded areas represent 95% confidence intervals (n = 2000 bootstraps each).

The number of aphids consumed by *H. axyridis* was affected by aphid density, aphid distribution, and the interaction between aphid density and aphid distribution when *A. gifuensis* was present (Table 2). In the heterospecific enemy combination, more aphids were consumed by *H. axyridis* in the aggregate treatment than in the uniform treatment when aphid density was between 50 and 230 aphids (Figure 2a). As for *A. gifuensis*, there was a significant effect of aphid density on the number of aphids parasitized by female *A. gifuensis* when *H. axyridis* was present (Table 2). When *H. axyridis* occurred, functional response of *A. gifuensis* overlapped across all prey densities between aggregate and uniform treatment (Figure 2b).

**Figure 3:**
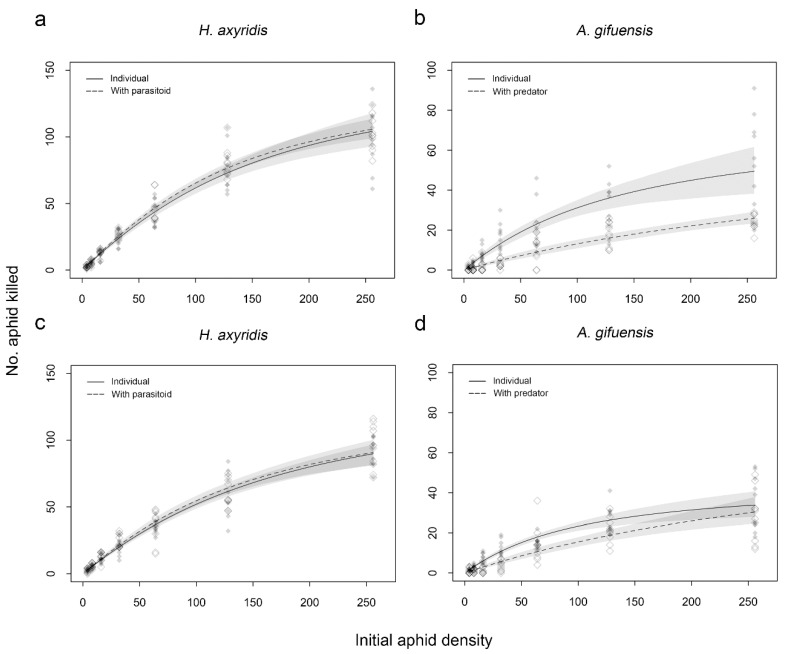
Functional response of single *Harmonia axyridis* or *Aphidius gifuensis* and the treatment when *H. axyridis* or *A. gifuensis* sharing the experimental unit with heterospecific enemy to densities of *Myzus persicae* under aggregate treatment (**a**,**b**) or uniform treatment (**c**,**d**). Shaded areas are bootstrapped 95% confidence intervals (n = 2000 bootstraps each).

Functional response curves were overlapped between the treatment where *H. axyridis* was alone and the treatment where *H. axyridis* was sharing the experimental patch with *A. gifuensis* for aggregate and uniform treatments, respectively (Figure 3a,c). Inversely, differences in functional response between the treatment where *A. gifuensis* was alone and the treatment where *A. gifuensis* was sharing the experimental patch with *H. axyridis* were detected in the aggregation or uniform treatment at aphid densities below 150, respectively (Figure 3b,d).

## 4. Discussion

In this study, aphid killed rates declined with increasing aphid densities in all cases. Logistic regression analysis indicated that the data from all treatments could fit the type II functional response curve statistically. The type II functional response is common in aphid predators and parasitoids [51,52]. For type II functional response, the unstable enemy-pest dynamic is likely to occur because the predation/parasitism rate would decrease with increasing prey density. Therefore, predators or parasitoids that exhibit type II functional response often cause prey extinction at low densities, but do not affect the prey populations at high prey densities [21,53]. When predators or parasitoids with type II response are applied in biological control systems, a high enemy-pest ratio is necessary to achieve effective pest suppression [2].

In this study, we found that both the killed rates of single *H. axyridis* and *A. gifuensis* were affected by prey distribution. Our results are similar to that of Feng et al. [44]. Single *H. axyridis* or *A. gifuensis* exhibited higher functional response curves in the aggregate treatment than in the uniform treatment at aphid densities between 40–180 or 70–170, respectively. The possible explanation is that *H. axyridis* or *A. gifuensis* may have enough searching time in a 24-h foraging period, enabling *H. axyridis* or *A. gifuensis* to encounter, consume or parasitize more aphids when aphid densities were low. With increasing aphid density, both *H. axyridis* and *A. gifuensis* may reveal area-restricted foraging behavior in the aggregate treatment like other predators and parasitoids [30,53]. However, when *H. axyridis* or *A. gifuensis* are foraging in patches with prey uniformly distributed, they may move more frequently between patches and spend more time in their searching process, and thus decrease aphid predation/parasitism. However, the majority of insect predators are digestion-limited, and the digestion process could affect their foraging efficiency [44]. For *H. axyridis*, this means that they digest prey slower than they handle them. Therefore, aphid consumption by *H. axyridis* in two aphid distributions did not differ significantly when aphid densities were above 180. As for *A. gifuensis*, the parasitism capacity may be limited by the parasitoid egg number in their body like other parasitoids [54], which did not increase parasitism efficiency with aphid density increased to around 170 in either aphid distributions.

Prey distribution could affect the functional response of *H. axyridis* when *A. gifuensis* was present. The reasons for the results may be similar to the single *H. axyridis* treatment. However, the number of aphids parasitized by *A. gifuensis* sharing the same experimental unit with *H. axyridis* was not affected by aphid distribution. These results differ from our initial hypothesis. Previous studies found that predators and parasitoids may reveal area-restricted foraging behavior in high prey quality patches [26,33,44], which may increase the antagonistic and intraguild interaction strength between *H. axyridis* and *A. gifuensis*. In our experiment, when *H. axyridis* was present, there was a trend towards increasing the number of aphids parasitized by *A. gifuensis* in the uniform treatment compared with aggregate treatment, but the differences were not significant. This might be due to the complementary resource use and partition resources by predators and parasitoids [55,56]. In resource partitioning, natural enemy species consume different subpopulations of prey so that a greater proportion of the total prey populations can be exploited by multispecies communities [6,57,58,59,60]. In the present study, *H. axyridis* and *A. gifuensis* maybe partitioning resources across other, unexplored niche axes, and thus did not affect the parasitism efficiency across all aphid densities in the aggregate treatment compared with uniform treatment. Future studies are needed to consider this possibility and explore the mechanisms.

The number of aphids consumed by *H. axyridis* was not affected by the presence of *A. gifuensis* compared with single *H. axyridis* across all aphid densities under two aphid distributions. In fact, the estimated handling times and attack rates of *H. axyridis* were similar, independently of the presence of *A. gifuensis*. Previous studies found that female *H. axyridis* did not exhibit any preference for unparasitized *Aphis glycines* Matsumura and aphids parasitized by *Aphelinus certus* Yasnosh [61]. Accordingly, it is possible that *H. axyridis* did not exhibit a preference between unparasitized *M. persicae* and parasitized aphids. Nevertheless, parasitoid progeny survivorship was higher in the treatment where *A. gifuensis* was alone in the aggregate treatment or at aphid densities below 150 in the uniform treatment than in the treatment when *A. gifuensis* was sharing the same unit with *H. axyridis*. This means that intraguild interactions occurred between *H. axyridis* and *A. gifuensis*. Previous studies found that predators would feed on more prey with increasing levels of prey aggregation because they prefer patches with higher prey densities [30,53]. This may increase the predation rate of *H. axyridis* on parasitized aphids when all prey were aggregated in one plant. On the aphid uniform treatment, *H. axyridis* could have enough searching time in the 24-h exposure period when aphid densities were low. With aphid density increasing, *H. axyridis* may move more frequently between patches and spend more time on foraging prey, and then the encounter probability between the predator and parasitized aphid starts to drop off. This may increase the possibility of *A. gifuensis* progeny survivorship in the uniform treatment when aphid densities were high.

## 5. Conclusions

In the current study, density dependent predation rate of *H. axyridis* was affected by aphid distribution, but not influenced by heterospecific interactions. The parasitism rate of *A. gifuensis* was affected by aphid distribution, and by heterospecific interactions in both the aggregate and uniform treatments. Therefore, to optimize the management efficiency of *M. persicae*, the combined use of *H. axyridis* and *A. gifuensis* would be appropriate when *M. persicae* is nearly uniformly distributed under relative high density. Previous studies found that multiple natural enemies could show an additive control efficiency when intraguild predation did not occur [62]. To optimize the efficiency of pest suppression when using multiple enemies, it is essential to evaluate the pest density thresholds at which mortality caused by two types of natural enemies changes from nonadditive to additive under different prey distributions. However, given the effect of laboratory rearing on the parasitoid foraging efficiency [63], heterospecific interactions of these two natural enemies under field conditions may differ from the laboratory results. Therefore, more studies are needed to investigate the effects of various types of factors that might affect the interactions and control efficiency of *A. gifuensis* or *H. axyridis*. In addition, further long-term studies are required to assess how *H. axyridis* may affect the long-term population abundance and dynamics of *A. gifuensis* under field conditions.

## Figures and Tables

**Table 1:** Significance levels from linear-term logistic regression of the number of *Myzus persicae* killed by single *Harmonia axyridis* or *Aphidius gifuensis*, individual *H. axyridis* or *A. gifuensis* in heterospecific combination at 24 h in two aphid distributions, and functional response parameters for Rogers random predator equation (*a* and *T*_h_ with mean, 95% CI).

Treatments (E: Estimated)	Distribution	Linear Coefficient, *p*	Type of Response Fitted	Attack Rate *a* (Estimated with 95% CI)	*p* (Z Value)	Handling Time *T*_h_ (In Hour) (Estimated with 95% CI)	*p* (Z Value)
Single *H. axyridis*	Aggregate	−0.006, <0.0001	Type 2	1.590 (1.306–1.979)	<0.0001(22.843)	0.006 (0.005–0.008)	<0.0001(21.484)
	Uniform	−0.005, <0.0001	Type 2	1.145 (0.961–1.358)	<0.0001 (20.391)	0.007 (0.006–0.008)	<0.0001 (16.312)
Single *A. gifuensis*	Aggregate	−0.004, <0.0001	Type 2	0.645 (0.465–0.890)	<0.0001 (14.956)	0.013 (0.007–0.021)	<0.0001 (13.047)
	Uniform	−0.005, <0.0001	Type 2	0.541 (0.424–0.678)	<0.0001 (12.610)	0.022 (0.015–0.029)	<0.0001 (13.176)
*H. axyridis* in heterospecific combination	Aggregate	−0.007, <0.0001	Type 2	1.908 (1.629–2.263)	<0.0001 (22.838)	0.007 (0.006–0.008)	<0.0001 (24.802)
	Uniform	−0.005, <0.0001	Type 2	1.317 (1.024–1.642)	<0.0001 (20.096)	0.007 (0.006–0.009)	<0.0001 (18.197)
*A. gifuensis* in heterospecific combination	Aggregate	−0.002, <0.0001	Type 2	0.177 (0.129–0.249)	<0.0001 (11.469)	0.015 (0.006–0.024)	<0.0001 (4.5899)
	Uniform	−0.002, <0.0001	Type 2	0.213 (0.162–0.312)	<0.0001 (12.402)	0.013 (0.002–0.024)	<0.0001 (5.1391)

**Table 2:** Generalized linear mixed model (GLMM) testing the effects of fixed factors on the number of aphids consumed/parasitized by single *Harmonia axyridis* or *Aphidius gifuensis*, individual *H. axyridis* or *A. gifuensis* in heterospecific combination (n = 70 for each assay). Observation level factor (tested enemies) were included as random effects. Seven levels of aphid density (4, 8, 16, 32, 64, 128, or 256 aphids per cage) were provided to each enemy assay.

Predator Treatments	Model Factors	Estimated	SE	*t*	*p*
Single *H. axyridis*	Aphid density	0.399	0.018	22.693	**<0.0001**
	Aphid distribution	−2.149	2.104	−1.021	**<0.001**
	Aphid density × aphid distribution	−0.047	0.019	−2.493	**0.013**
Single *A. gifuensis*	Aphid density	0.195	0.013	14.587	**<0.0001**
	Aphid distribution	−0.128	1.730	−0.074	**<0.001**
	Aphid density × aphid distribution	−0.066	0.015	−4.285	**<0.0001**
*H. axyridis* in heterospecific combination	Aphid density	0.405	0.016	24.962	**<0.0001**
	Aphid distribution	−2.676	1.742	−1.536	**<0.0001**
	Aphid density × aphid distribution	−0.045	0.016	−2.887	**0.004**
*A. gifuensis* in heterospecific combination	Aphid density	0.099	0.008	11.681	**<0.0001**
	Aphid distribution	0.272	1.044	0.261	0.056
	Aphid density × aphid distribution	0.017	0.009	1.831	0.067

Bold letters indicate significant differences between treatments (*p* < 0.05).

## References

[1] Bloemhard C.M.J., Ramakers P.M.J. (2008). Strategies for aphid control in organically grown sweet pepper in the Netherlands. IOBC/WPRS.

[2] van Lenteren J.C. (2012). The state of commercial augmentative biological control: Plenty of natural enemies, but a frustrating lack of uptake. BioControl.

[3] Barratt B.I.P., Moran V.C., Bigler F., van Lenteren J.C. (2018). The status of biological control and recommendations for improving uptake for the future. BioControl.

[4] Straub C.S., Snyder W.E. (2008). Increasing enemy biodiversity strengthens herbivore suppression on two plant species. Ecology.

[5] Gontijo L.M., Beers E.H., Snyder W.E. (2015). Complementary suppression of aphids by predators and parasitoids. Biol. Control.

[6] Snyder W.E. (2019). Give predators a complement: Conserving natural enemy biodiversity to improve biocontrol. Biol. Control.

[7] Snyder W.E., Ives A.R. (2003). Interactions between specialist and generalist natural enemies: Parasitoids, predators, and pea aphid biocontrol. Ecology.

[8] Michaelides G., Sfenthourakis S., Pitsillou M., Seraphides N. (2018). Functional response and multiple predator effects of two generalist predators preying on *Tuta absoluta* eggs. Pest Manag. Sci..

[9] Schmitz O.J. (2009). Effects of predator functional diversity on grassland ecosystem function. Ecology.

[10] Frago E., Godfray H.C.J. (2014). Avoidance of intraguild predation leads to a long-term positive trait-mediated indirect effect in an insect community. Oecologia.

[11] Rocca M., Rizzo E., Greco N., Sánchez N. (2017). Intra- and interspecific interactions between aphidophagous ladybirds: The role of prey in predator coexistence. Entomol. Exp. Appl..

[12] Lang B., Rall B.C., Brose U. (2012). Warming effects on consumption and intraspecific interference competition depend on predator metabolism. J. Anim. Ecol..

[13] Messelink G.J., Sabelis M.W., Janssen A., Larramendy M.L., Soloneski S. (2012). Generalist predators, food web complexities and biological pest control in greenhouse crops. Integrated Pest Management and Pest Control-Current and Future Tactics.

[14] Vanaclocha P., Papacek D., Monzó C., Verdú M.J., Urbaneja A. (2013). Intra-guild interactions between the parasitoid *Aphytis lingnanensis* and the predator *Chilocorus circumdatus*: Implications for the biological control of armoured scales. Biol. Control.

[15] Martinou A.F., Raymond B., Milonas P.G., Wright D.J. (2010). Impact of intraguild predation on parasitoid foraging behaviour. Ecol. Entomol..

[16] Lipcius R.N., Hines A.H. (1986). Variable functional responses of a marine predator in dissimilar homogeneous microhabitats. Ecology.

[17] Fernández-arhex V., Corley J.C. (2003). The functional response of parasitoids and its implications for biological control. Biocontrol Sci. Technol..

[18] Okuyama T. (2013). On selection of functional response models: Holling’s models and more. BioControl.

[19] Dick J.T.A., Alexander M.E., Jeschke J.M., Ricciardi A., MacIsaac H.J., Robinson T.B., Kumschick S., Weyl O.L.F., Dunn A.M., Hatcher M.J. (2014). Advancing impact prediction and hypothesis testing in invasion ecology using a comparative functional response approach. Biol. Invasions.

[20] Solomon M.E. (1949). The natural control of animal populations. J. Anim. Ecol..

[21] Murdoch W.W., Oaten A. (1975). Predation and population stability. Adv. Ecol. Res..

[22] Holling C.S. (1959). Some characteristics of simple types of predation and parasitism. Can. Entomol..

[23] Holling C.S. (1966). The functional response of invertebrate predators to prey density. Mem. Ent. Soc. Can..

[24] Verheggen F.J., Vogel H., Vilcinskas A. (2017). Behavioral and immunological features promoting the invasive performance of the harlequin ladybird *Harmonia axyridis*. Front. Ecol. Evol..

[25] Ritchie M.E. (1998). Scale-dependent foraging and patch choice in fractal environments. Evol. Ecol..

[26] Yazdani M., Keller M. (2015). Mutual interference in *Dolichogenidea tasmanica* (Cameron) (Hymenoptera: Braconidae) when foraging for patchily-distributed light brown apple moth. Biol. Control.

[27] Hassell M.P., May R.M. (1973). Stability in insect host-parasite models. J. Anim. Ecol..

[28] Ives A.R. (1992). Continuous-time models of host-parasitoid interactions. Am. Nat..

[29] Wajnberg É. (2006). Time allocation strategies in insect parasitoids: From ultimate predictions to proximate behavioral mechanisms. Behav. Ecol. Sociobiol..

[30] Kareiva P. (1990). Population dynamics in spatially complex environments: Theory and data. Philos. Trans. R. Soc. B.

[31] Godfray H.C.J. (1994). Host location. Parasitoids: Behavioral and Evolutionary Ecology.

[32] Zhou H., Yu Y., Tan X., Chen A., Feng J. (2014). Biological control of insect pests in apple orchards in China. Biol. Control.

[33] Hossie T.J., Murray D.L. (2016). Spatial arrangement of prey affects the shape of ratio-dependent functional response in strongly antagonistic predators. Ecology.

[34] Wang S.Y., Chi H., Liu T.X. (2016). Demography and parasitic effectiveness of *Aphelinus asychis* reared from *Sitobion avenae* as a biological control agent of *Myzus persicae* reared on chili pepper and cabbage. Biol. Control.

[35] Xia P., Liu Y., Fan J., Tan J. (2015). Geostatistical analysis on distribution dynamics of *Myzus persicae* (Sulzer) in flue-cured tobacco field. J. Appl. Ecol..

[36] Wang S., Zhang R.Z., Zhang F. (2007). Research progress on biology and ecology of *Harmonia axyridis* Pallas (Coleoptera: Coccinellidae). J. Appl. Ecol..

[37] Pan M.Z., Liu T.X. (2014). Suitability of three aphid species for *Aphidius gifuensis* (Hymenoptera: Braconidae): Parasitoid performance varies with hosts of origin. Biol. Control.

[38] Pan M.Z., Wang L., Zhang C.Y., Zhang L.X., Liu T.X. (2017). The influence of feeding and host deprivation on egg load and reproduction of an aphid parasitoid, *Aphidius gifuensis* (Hymenoptera: Braconidae). Appl. Entomol. Zool..

[39] Koch R.L. (2003). The multicolored Asian lady beetle, *Harmonia axyridis*: A review of its biology, uses in biological control, and non-target impacts. J. Insect Sci..

[40] Berkvens N., Bonte J., Berkvens D., Deforce K., Tirry L., De Clercq P., Roy H.E., Wajnberg E. (2007). Pollen as an alternative food for *Harmonia axyridis*. From Biological Control to Invasion: The Ladybird Harmonia axyridis as a Model Species.

[41] Ohta I., Ohtaishi M. (2004). Fertility, longevity and intrinsic rate of increase of *Aphidius gifuensis* Ashmead (Hymenoptera: Braconidae) on the green peach aphid, *Myzus persicae* (Sulzer) (Homoptera: Aphididae). Appl. Entomol. Zool..

[42] Lee J.H., Kang T.J. (2004). Functional response of *Harmonia axyridis* (Pallas) (Coleoptera: Coccinellidae) to *Aphis gossypii* Glover (Homoptera: Aphididae) in the laboratory. Biol. Control.

[43] Seko T., Miura K. (2008). Functional response of the lady beetle *Harmonia axyridis* (Pallas) (Coleoptera: Coccinellidae) on the aphid *Myzus persicae* (Sulzer) (Homoptera: Aphididae). Appl. Entomol. Zool..

[44] Feng Y., Zhou Z.X., An M.R., Yu X.L., Liu T.X. (2018). The effects of prey distribution and digestion on functional response of *Harmonia axyridis* (Coleoptera: Coccinellidae). Biol. Control.

[45] Feng Y., Zhou Z.X., An M.R., Li Y.D., Liu Z.G., Wang L.L., Ren J.Z., Liu T.X. (2018). Conspecific and heterospecific interactions modify the functional response of *Harmonia axyridis* and *Propylea japonica* to *Aphis citricola*. Entomol. Exp. Appl..

[46] Lü X.K., Xu X., Ma L., Liu Q., Chen G.H., Li Q. (2013). Study on characteristics and dynamics of arthropod community in corn field of Zhaotong, Yunnan. J. Environ. Entomol..

[47] Core Team R. (2019). R: A Language and Environment for Statistical Computing.

[48] Juliano S., Scheiner S.M., Gurevitch J. (2001). Non-linear curve fitting: Predation and functional response curves. Design and Analysis of Ecological Experiments.

[49] Rogers D. (1972). Random search and insect population models. J. Anim. Ecol..

[50] Pritchard D., Barrios-O’Neill D., Bovy H., Paterson R., Pritchard M.D. (2017). Frair: Functional response analysis in R. R Package Version 0.4. http://CRAN.R-project.org/package=frair.

[51] Frewin A.J., Xue Y., Welsman J.A., Broadbent A.B., Schaafsma A.W., Hallett R.H. (2010). Development and parasitism by *Aphelinus certus* (Hymenoptera: Aphelinidae), a parasitoid of *Aphis glycines* (Hemiptera: Aphididae). Environ. Entomol..

[52] Atlıhan R., Kaydan M.B., Yarımbatman A., Okut H. (2010). Functional response of the coccinellid predator *Adalia fasciatopunctata reveliereito* walnut aphid (*Callaphis juglandis*). Phytoparasitica.

[53] Hassell M.P. (1978). Non-Random Search. The Dynamics of Arthropod Predator-Prey Systems.

[54] Dieckhoff C., Theobald J.C., Wäckers F.L., Heimpel G.E. (2014). Egg load dynamics and the risk of egg and time limitation experienced by an aphid parasitoid in the field. Ecol. Evol..

[55] Borer E.T., Murdoch W.W., Swarbrick S.L. (2004). Parasitoid coexistence: Linking spatial field patterns with mechanism. Ecology.

[56] Frago E. (2016). Interactions between parasitoids and higher order natural enemies: Intraguild predation and hyperparasitoids. Curr. Opin. Insect Sci..

[57] Loreau M., Naeem S., Inchausti P., Bengtsson J., Grime J.P., Hector A., Hooper D.U., Huston M.A., Raffaelli D., Schmid B. (2001). Biodiversity and ecosystem functioning: Current knowledge and future challenges. Science.

[58] Tilman D., Reich P.B., Knops J., Wedin D., Mielke T., Lehman C. (2001). Diversity and productivity in a long-term grassland experiment. Science.

[59] Casula P., Wilby A., Thomas M.B. (2006). Understanding biodiversity effects on prey in multi-enemy systems. Ecol. Lett..

[60] Greenop A., Woodcock B.A., Wilby A., Cook S.M., Pywell R.F. (2018). Functional diversity positively affects prey suppression by invertebrate predators: A meta-analysis. Ecology.

[61] Xue Y., Bahlai C.A., Frewin A., McCreary C.M., Marteaux L.E.D., Schaafsma A.W., Hallett R.H. (2012). Intraguild predation of the aphid parasitoid *Aphelinus certus* by *Coccinella septempunctata* and *Harmonia axyridis*. BioControl.

[62] Ferguson K.I., Stiling P. (1996). Non-additive effects of multiple natural enemies on aphid populations. Oecologia.

[63] Bautista R.C., Harris E.J. (1997). Effect of insectary rearing on host preference and oviposition behavior of the fruit fly parasitoid *Diachasmimorpha longicaudata*. Entomol. Exp. Appl..

